# One Standard for All: Uniform Scale for Comparing Individuals and Groups in Hierarchical Bayesian Evidence Accumulation Modeling

**DOI:** 10.5334/joc.394

**Published:** 2024-08-16

**Authors:** Rotem Berkovich, Nachshon Meiran

**Affiliations:** 1 Ben Gurion University of the Negev, Beer-Sheva, Israel

**Keywords:** Evidence Accumulation Models, Linear Ballistic Accumulation model, Bayesian hierarchical models

## Abstract

In recent years, a growing body of research uses Evidence Accumulation Models (EAMs) to study individual differences and group effects. This endeavor is challenging because fitting EAMs requires constraining one of the EAM parameters to be equal for all participants, which makes a strong and possibly unlikely assumption. Moreover, if this assumption is violated, differences or lack thereof may be wrongly found. To overcome this limitation, in this study, we introduce a new method that was originally suggested by van Maanen & Miletić (2021), which employs Bayesian hierarchical estimation. In this new method, we set the scale at the population level, thereby allowing for individual and group differences, which is realized by *de facto* fixing a population-level hyper-parameter through its priors. As proof of concept, we ran two successful parameter recovery studies using the Linear Ballistic Accumulation model. The results suggest that the new method can be reliably used to study individual and group differences using EAMs. We further show a case in which the new method reveals the true group differences whereas the classic method wrongly detects differences that are truly absent.

## Introduction

In recent years, there is a growing body of research that uses Evidence Accumulation Models (EAMs) to reveal the cognitive mechanism that accounts for experimental effects (Forstmann et al., 2016), and these models are recently also extensively used to study individual and group differences (Pleskac et al., 2019; Schubert & Frischkorn, 2020; Sripada & Weigard, 2021). EAMs are a family of models all sharing the assumption that the representation of stimuli in the central nervous system is noisy, and a decision is made after accumulating successive samples from this noisy representation until a sufficient amount of evidence has been obtained and a decision criterion has been reached (Ratcliff & Smith, 2004). Core parameters in EAM include the across-trials mean rate of evidence accumulation- drift-rate (*v*), and the standard deviation the drift rate (*sv*).

However, using EAMs turns out to be challenging (van Maanen & Miletić, 2021). Specifically, when fitting EAMs, one needs to constrain at least one parameter that will set the scale for the remaining parameters. This requirement arises since the parameters of the EAMs represent latent variables, which require that their units be defined.

More specifically, the mean rate of evidence accumulation, the drift-rate, is expressed in terms of units-of-evidence per second. While time (measured in seconds) is not latent, the latent “unit of evidence” must somehow be defined. A common practice in fitting the Linear Ballistic Accumulator model (LBA; Brown & Heathcote, 2008), which is a type of EAM, is to set the unit such that one of the parameters is fixed at unity and sets the scale for the remining parameters. Analogous practices are used when fitting other EAMs such as the Drift-Diffusion Model (DDM, Ratcliff & McKoon, 2008). This practice is also not unique to EAMs. For example, in Signal-Detection Theory (Macmillan & Creelman, 2004), the unit which is used to define sensitivity is often set to be the standard deviation of the noise. In other words, when comparing individuals/conditions, an implicit assumption is made that the parameter being fixed at the same value for all participants, such as the standard deviation of the noise is equal (to unity) across participants.

The problematics associated with this practice can be illustrated with the concept of distance. If one arbitrarily defines the unit for distance as the unit that is being used in a given country, one may wrongly conclude that two US cities that are 200 miles apart are as distant as two cities in France that are 200 km apart, for example. This is in fact analogous to how the unit is defined when fitting EAM models: such that the unit is defined per participant (participant being analogous to country, in this example). The proper comparison requires adopting a constant unit that applies across countries/participants, of course.

Setting the scale in this way (e.g., fixing a parameter at some value, such as unity) implies assuming lack of meaningful individual and group differences in the parameter which is used for scale setting. This is a powerful assumption that might be wrong. For example, one might decide to use *sv* to set the scale, but *sv* may be said to represent the degree of noise, and brain recordings suggest that there are important individual and group differences in neural noise (Dinstein et al., 2015). The implication of violating the assumption regarding lack of meaningful differences in the fixed parameter concerns erroneous individual/experiment differences (or lack thereof) in the estimate model parameters. For instance, imagine that two participants are truly different from one another in *sv*, but are truly *not* different from one another in the free (estimated) parameter (*v*). Since parameter estimation is commonly made by fixing a parameter such as *sv* at some value, it is possible to find individual differences or an experimental effect in a parameter (e.g., *v*) where such differences are absent, and failing to find them in a parameter (*sv*) where such differences exist. A reviewer suggested an alternative by which instead of fixing *sv* across participants, one may fix another parameter such as the boundary, and employ an experimental manipulation (e.g., accuracy emphasis) that likely eliminates any individual or group differences. We argue, however, this approach still runs some risk since it cannot be fully assured that the individual/group differences were truly eliminated by the manipulation. The method suggested in this work overcomes the problem much more effectively.

Two solutions to the aforementioned problem were suggested by van Maanen & Miletić (2021). The first of these two solutions was also empirically demonstrated by these authors. It consists of showing that when one interprets the results in terms of how parameters are related relative to one another results in a consistent interpretation across different parameter constraints. van Maanen & Miletić ‘s second solution was just suggested and was not empirically tested. The proposal was to fit the data with a Bayesian hierarchical model. In the second solution, since participants are nested within a group, it is possible to instead of defining the unit by constraining the individual-level parameters (e.g., all participants’ *sv* values are 1) to constrain the group level parameter (e.g., the population mean *sv* = 1). This constraint is accomplished through the setting of the Bayesian priors. This method sets the scale (unit = population-level mean *sv*) while still allowing for individual and group differences in (e.g.) *sv*. It seems to us that the second (not yet tested) solution is more effective because it involves eliminating the source of the problem. *Therefore, in the current study we adopt this second solution and suggest a practical approach to implement it. We validate the new method using a simple EAM: The LBA, in its Bayesian hierarchical estimation version (**Lin & Strickland, 2020**)*. The next section provides a short description of the LBA.

### LBA

In the LBA, each choice alternative is associated with an evidence accumulator. The initial amount of evidence in each accumulator prior to evidence accumulation is determined by the *starting point* parameter, which defines the range (0-*starting point*) from which the amount is sampled in each trial. In each trial, the drift rate is also sampled from a normal distribution with mean drift rate (*v*) and a standard deviation of the drift rate (*sv*). The LBA is acknowledged to be simplified because it assumes that evidence accumulation rate remains constant in a given trial (hence, “Linear Ballistic”). A decision is reached once the amount of evidence in an accumulator crosses the *boundary, b*, which is sometimes estimated indirectly through *B*, where *b = starting point +B*. The last parameter is non-decision time (*t0*) expressed in sec and describing the duration of the non-decisional processes such as early feature extraction and motor preparation.

When applying the model using the Bayesian hierarchical method, one estimates the parameters for each individual, separately, but constrains this estimation with a population level distribution. The process of model fitting starts with the definition of the model which includes the determination of the free (to be estimated) parameters and the fixed parameter that sets the scale. Following, one needs to specify two types of priors: base- level priors, which are the priors for each parameter at the individual participant’s level (typically, all participants’ parameters have the same priors), and hyper level priors which are the priors for the parameters that characterize the population. Estimation proceeds by posterior sampling using Markov Chains Monte Carlo (MCMC) method. Posterior sampling adequacy is assessed using the Potential Scale Reduction Factor (PSRF), that should fall below 1.1 (Brooks & Gelman, 1998).

### The new method

In the new method, we *de facto* fixed *sv* at the *population* level. This was done indirectly using the hyper- level Bayesian priors. More specifically, we defined the *Mu* (population level mean) prior for *sv*, using a Beta distribution with α = β = 1 (creating a uniform distribution) and a tiny range from .999 to 1.001. This method *de facto* fixes the mean of the population *sv* at about 1, and thus sets the scale (one unit of evidence is equal to the population-level *sv*), but still allows for individual and group differences in *sv*. In the present work, we report two parameter recovery studies, providing the necessary proof of concept for the new method. In each parameter-recovery study, we simulated data using pre-determined parameter values, and then estimated the parameter values with the model, and checked whether the estimates are close enough to the pre-determined values.

To summarize, the goal of this study was to find a credible implementation of the notion to fix one parameter at the population level, which allows the EAM (here, LBA) to investigate individual differences or experimental effects while not being exposed to the aforementioned risks.

## Study 1

The goal of Study 1 was to check whether the parameter recovery, using the new method, is successful.

### Method

**Figure 1:**
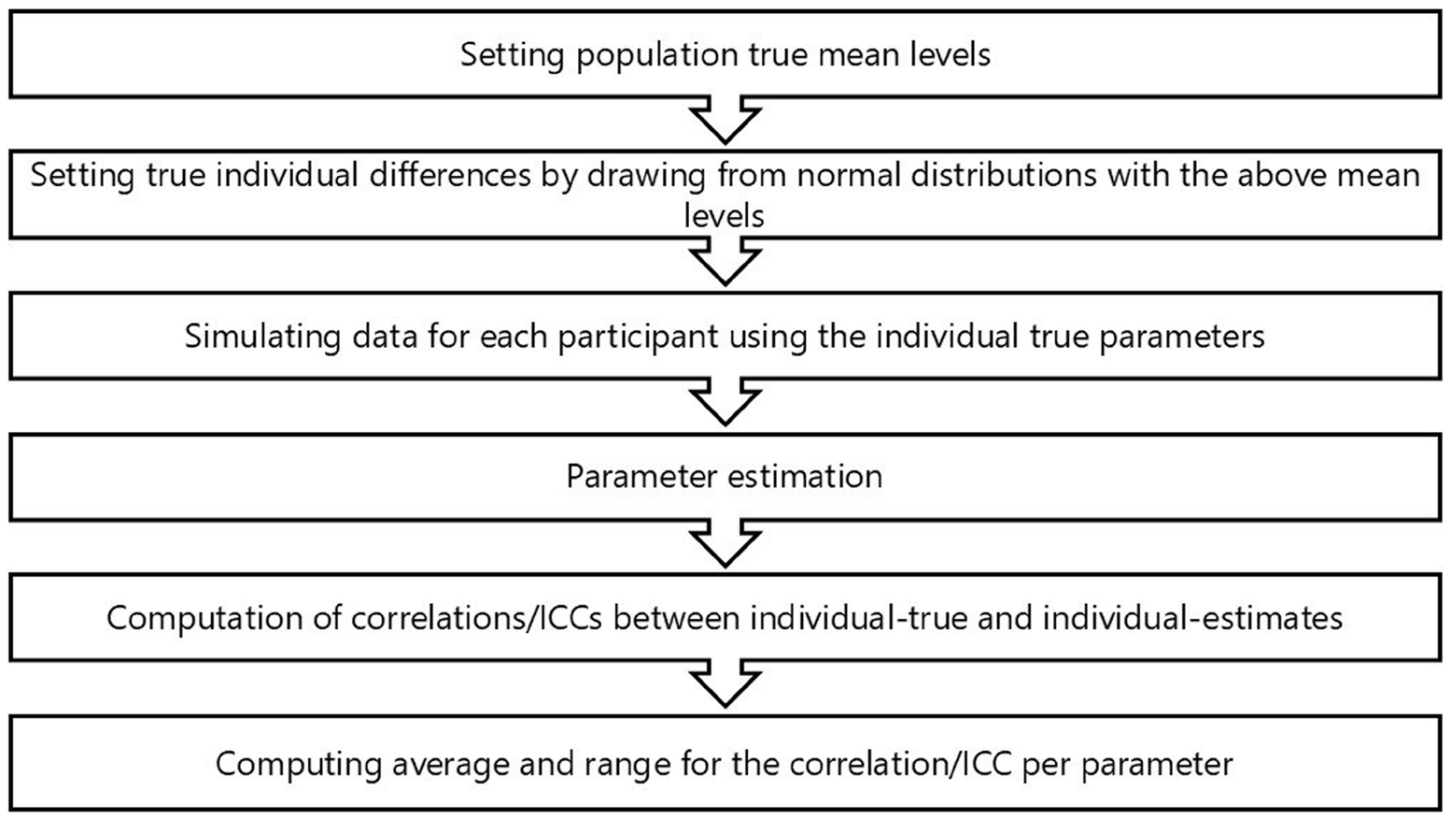
Summary of the steps involved in Study 1.

The steps involved in Study 1 are summarized in Figure 1. We first simulated 21 data sets, that differed from one another in the combinations of mean (across participants) parameter values (Table 1). Based on pervious empirical work (Berkovich & Meiran, 2023; Berkovich & Meiran, 2024; Givon et al., 2023), the range of parameter values is realistic for the type of tasks that we study in our lab. Given the fact that each model fitting takes a very long time (just to give an idea, a single fitting for the 21 models took about one month to converge, despite using massive parallel processing), we limited the number of mean-combinations by not changing the *starting point* mean value, which was fixed across simulations at 2. Additionally, since research questions that concern *t0* are not the focus of our studies, the value of *t0* for all ”participants” in all models was set to 0.3, and its recovery was accordingly not tested. Not changing t0 and the fact that we used a particular range of parameter values consist of a limitation of the current study.

**Table 1:** Population mean value in the 21 models from Study 1. v. TRUE and v. FALSE represent the mean drift rate for the correct and incorrect response, respectively.


*STARTING POINT*	*BOUNDARY*	*V. TRUE*	*V. FALSE*	*sv*

2	2.3	2.3	0.6	1

2	2.5	2.3	0.6	1

2	2.7	2.3	0.6	1

2	2.3	2.5	0.6	1

2	2.5	2.5	0.6	1

2	2.7	2.5	0.6	1

2	2.3	2.7	0.6	1

2	2.5	2.7	0.6	1

2	2.7	2.7	0.6	1

2	2.3	2.3	0.8	1

2	2.5	2.3	0.8	1

2	2.7	2.3	0.8	1

2	2.3	2.5	0.8	1

2	2.5	2.5	0.8	1

2	2.7	2.5	0.8	1

2	2.3	2.7	0.8	1

2	2.5	2.7	0.8	1

2	2.7	2.7	0.8	1

2	2.3	2.3	1	1

2	2.5	2.3	1	1

2	2.7	2.3	1	1


To simulate participants’ data, we first determined the “true” individual parameter values by drawing from normal distributions, with means as defined in Table 1, and with a standard deviation being 0.6 for *starting point* and *boundary*, 0.8 for *v. true* and *v. false*, and 0.4 for *sv*. Randomly drawn values were corrected if they fell outside an allowable range. The lower bound for *starting point, boundary* and *v. false* was 0.05, for *v. true* the lower bound was 1.3, and for *sv* the lower bound was 0.01. Additionally, if the difference between *v. true* and *v. false* in a specific model was smaller than 0.2 (favoring *v. true)*, then the value of *v. false* was replaced by the value of *v. true* minus 0.2. Using these “true” individual parameter values, we simulated 21 datasets, each comprising 100 “participants”, each with 1,000 “trials”.

Each such simulated dataset was then fitted using Bayesian hierarchical LBA. The priors for individual estimates were the mean values that were used for simulation with noise added. We added noise to the mean values by adding a value that was drawn from a normal distribution with *mean* = 0 and *SD* = 0.1. Adding noise makes the priors more realistic in the sense of being “in the neighborhood” of the true value but not being exactly at it.

The population level prior means were the same as the individual level prior means except for *sv* which was defined using (uniform) Beta distribution with α and β values set to 1 and range from 0.999 to 1.001. The Sigma prior (population standard deviation) was uninformative (meaning that it was de factor determined by the data alone and not by the priors) and was defined with a (uniform) Beta distribution, with α = β = 1 with a range of 0–3.

Posterior sampling was accomplished with a burn in period of 1,000 samples per sample-chain and actual sampling of 12,000 samples and thinning equal to 12, which means keeping each 12^th^ sample. We used the default number of chains: number of free parameters times three.

The results for each one of the 21 data-sets thus comprised of a set of “true” parameter values (those used to simulate the data) for each one of the 100 “participants” and estimated parameters for these “participants”. Of interest was the degree of correspondence between the “true” and estimated parameter values across participants in each one of the data-sets.

### Method for assessing parameter recovery

After the model has converged, we calculated PSRF for each participant (to assess model convergence). Then we computed the Pearson correlation and the ICC_2,1_ between the “true” parameter values and the (estimated) recovered parameter values. The logic is that if Pearson correlation and the ICC_2,1_ are high, we can deduce that the new method is credible and can be used to compare individuals/groups. This is true in the sense that if the LBA provides a reasonable approximation of the data generation process, then the estimated parameter values correctly represent that operation of this process. We used both Pearson correlation and ICC_2,1_ to allow us to inspect both the stability of the relative score (Pearson correlation) and the absolute agreement (ICC_2,1_). Since this procedure produced 21 correlations and 21 ICC’s, we averaged these values through Fisher’s Z-transformation. It is important to note that while we employed a Bayesian method for parameter estimation, the hypothesis testing was conducted using frequentist statistics. This approach was taken because we are not aware of a Bayesian method to test the ICC for significance. Consequently, we decided to utilize frequentist statistics for all our tests to maintain consistency.

### Results

Most of the models converged successfully, with PSRF falling below 1.1 for 95%–100% of the ”participants” in each model. Only in the case of one model, PSRF fell below 1.1 for only 19% of the “participants”. Since for some simulated participants, PSRF was unsatisfactory and fell above 1.1, we report the correlations and the ICC_2,1_s value after removing these “participants”. The parallel analysis that includes these “participants” did not change the picture too much and is reported in the Supplementary Materials.

**Figure 2:**
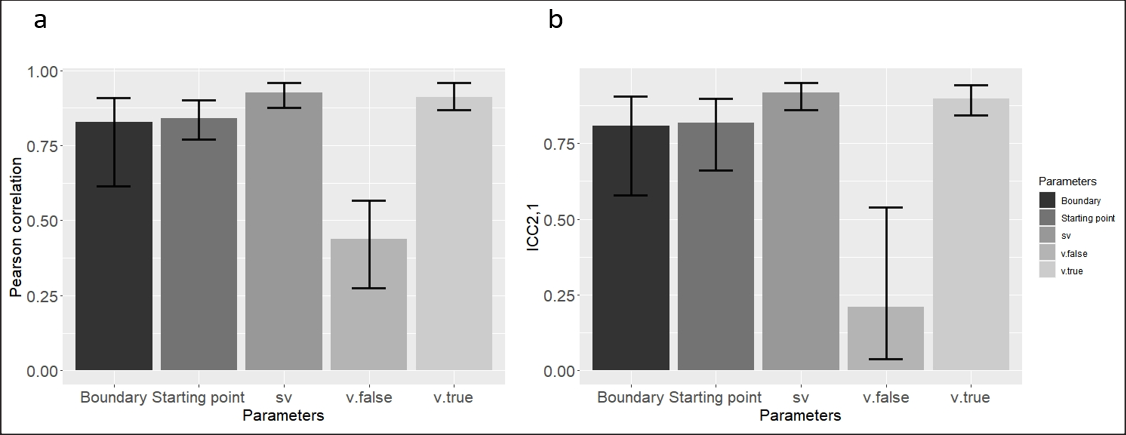
Mean **(a)** Pearson correlation, and **(b)** ICC2,1 Across the 21 Simulated Datasets (Means were computed through Fisher’s Z transformation), using the new method in Study 1. Error bars represent the lower and upper range of correlations from the 21 models.

We found that with the exception of *v.false*, all correlation and all ICC_2,1_s were significantly different from zero (*p* < 0.05). For the *v.false* its ICC_2,1_ was not significant in 8 out of the 21 models. The range of values is shown in Table 2. Additionally, the Pearson correlation results are shown in Figure 2a, and ICC_2,1_ results are shown in Figure 2b.

**Table 2:** Pearson correlation and ICC_2,1_ range and mean for all parameters using the new method in Study 1.


*PARAMETER*	*METHOD*	*LOWER BOUND*	*HIGHER BOUND*	*MEAN VALUE*

*Starting point*	Pearson cor.	0.770	0.900	0.840

*Boundary*	Pearson cor.	0.614	0.909	0.827

*v.true*	Pearson cor.	0.867	0.959	0.911

*v.false*	Pearson cor.	0.274	0.566	0.439

*sv*	Pearson cor.	0.875	0.958	0.924

*Starting point*	ICC_2,1_	0.660	0.897	0.818

*Boundary*	ICC_2,1_	0.578	0.905	0.806

*v.true*	ICC_2,1_	0.842	0.941	0.898

*v.false*	ICC_2,1_	0.038	0.540	0.209

*sv*	ICC_2,1_	0.861	0.950	0.917


It seems that all parameters, except for *v.false*, were recovered successfully at the individual (simulated) participant level, as reflected in both a reasonably high Pearson correlation and a reasonably high ICC_2,1_.

We suspected that *v.false* failed to recover successfully not because of the new method, but that it might be a disadvantage of the classic method as well. In order to test if this is indeed the case, we ran another parameter recovery study, using the exact same method as before, but this time we fixed *sv* = 1 for all participants, as customarily done (i.e., the “classic” method). The results of this parameter recovery are reported below.

This time we found that for all “participants” in all models, PSRF fell below 1.1, except for one “participant” in one model. This “participant” has been removed from the analysis and the results of the analyses that include this “participant” are reported in the Supplementary Materials.

**Figure 3:**
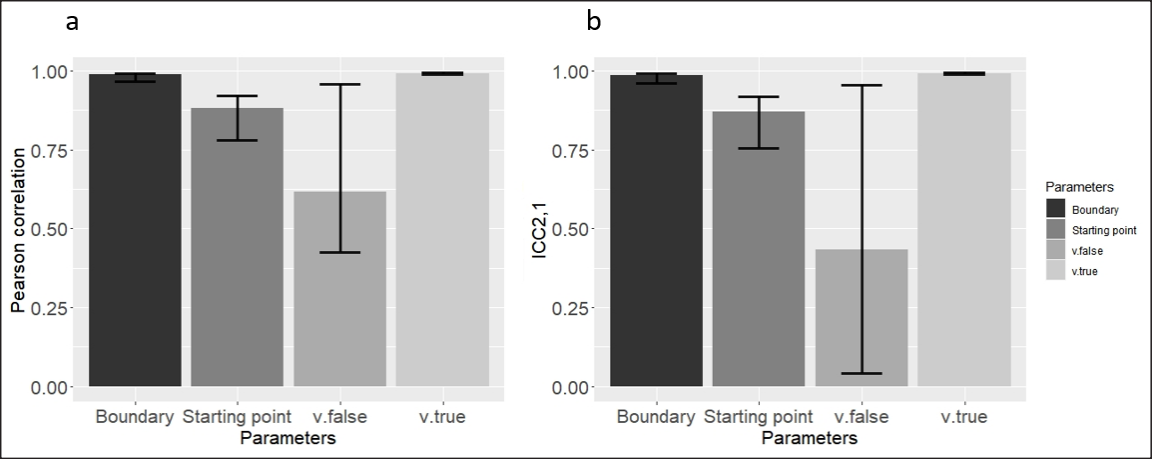
Mean **(a)** Pearson correlation, and **(b)** ICC2,1 Across the 21 Simulated Datasets (Means were computed through Fisher’s Z transformation), using the classic method in Study 1. Error bars represent the lower and upper range of correlations from the 21 models.

Once again, all Pearson correlations for all parameters were significantly different from zero (*p* < 0.05). This time, all ICC_2,1_ but that of *v.false* met this criterion. The results of this analysis are reported in Table 3 and Figure 3.

**Table 3:** Pearson correlation and ICC_2,1_ range and mean for all parameters using the classic method in Study 1.


*PARAMETER*	*METHOD*	*LOWER BOUND*	*HIGER BOUND*	*MEAN VALUE*

*Starting point*	Pearson cor.	0.780	0.920	0.881

*Boundary*	Pearson cor.	0.966	0.992	0.987

*v.true*	Pearson cor.	0.988	0.993	0.991

*v.false*	Pearson cor.	0.424	0.957	0.615

*Starting point*	ICC_2,1_	0.756	0.917	0.869

*Boundary*	ICC_2,1_	.960	0.992	0.984

*v.true*	ICC_2,1_	0.988	0.993	0.991

*v.false*	ICC_2,1_	0.0431	0.956	0.434


### Summary

The results of Study 1 demonstrate the applicability of the newly suggested method by a quite successful parameter recovery, including that of *sv*. However, in retrospect, we identified three shortcomings in our methodology. Specifically, (a) it is plausible that the successful parameter recovery was attained because the priors were unrealistically similar to the true population mean values. (b) It seems that the recovery by the new method, despite being successful, was less successful than when using the “classic” method. Additionally, (c) most real-world studies model at least one parameter as a function of groups or conditions, and this division is lacking in Study 1. (Nonetheless, individual differences in the parameter values were successfully captured). To address these shortcomings, we conducted Study 2, using a slightly different approach.

## Study 2

### Method

To address the first drawback, regarding the too-close-to-true priors, we adopted a different method to create the priors in which we sampled the priors from a uniform distribution that ranged two standard errors below and above the true parameter mean. We believe that this change more accurately mirrors real world applications, characterized by well-informed guessing, where researchers have extensive experience with the task at hand.

To address the second and third shortcoming, we wanted to demonstrate the most important feature of the new method which allows one to find true parameter differences without the risk of wrongly attributing differences to parameters that do not truly differ. Therefore, we simulated two groups of 50 “participants”, each. In the first group, *sv* mean value was 1.5, and in the second group, *sv* mean value was 0.5. The population mean values in the rest of the parameters were identical across the two groups: *Starting point* = 2, *Boundary* = 2.5, *v.true* = 2.5, *v.false* = 0.8, *t0* = 0.3. Then, we created the parameters for each participant, and the data, using the same method that was used in Study 1. To reveal the discrepancy between the new method and the classic method, we fitted the data twice: once using the new method, and a second time using the classic method. The idea was to demonstrate that the new method would reveal the true difference between groups (*sv*), but that the classic method might wrongly reveal non-existing groups differences in other parameters. This time, we also decided to assess model fit using the Deviance Information Creation (DIC; Spiegelhalter et al., 2014), in addition to just assessing parameter recovery success. Using DIC allowed us to compare between the new and the classic methods in terms of model fit, with a lower DIC indicating better fit. Following a reviewer’s comment, we add that the DIC may be inaccurate in complex models such as the LBA (Pooley & Marion, 2018). Nonetheless, DIC helped us (albeit tentatively) compare the two methods.

### Results

Modeling the data using both the classic and the new method was pretty successful in terms of convergence, with PSRF falling above 1.1 for only one “participant” in the classic method (2.45), and for three “participants” in the new method (2.01, 2.08, 2.23). As before, those “participants” were removed from further analyses, but the results of the analysis including them did not change the picture too much and are reported in the Supplementary Materials.

**Figure 4:**
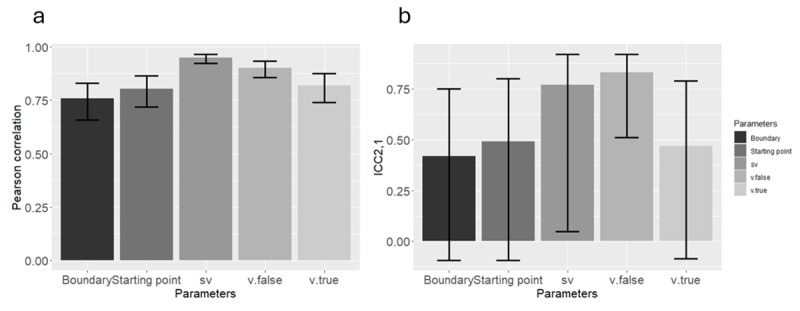
**(a)** Pearson correlation, and **(b)** ICC_2,1_, using the new method in Study 2. Error bars represent 95 percent confidence interval.

**Figure 5:**
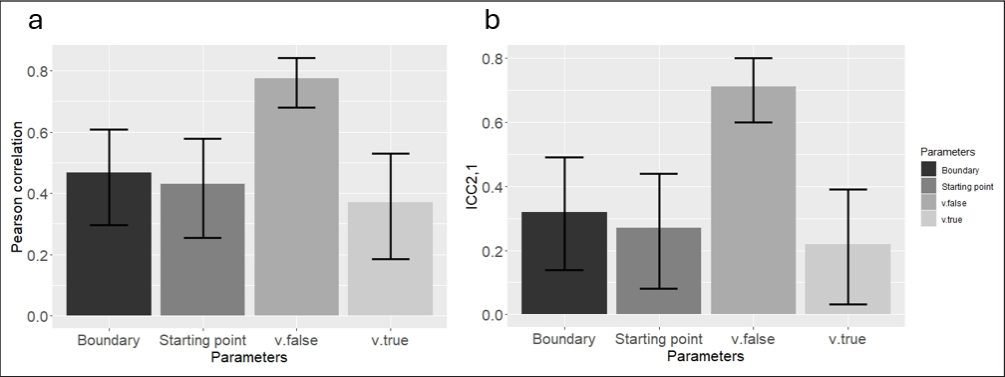
**(a)** Pearson correlation, and **(b)** ICC_2,1_, using the classical method in Study 2. Error bars represent 95 percent confidence interval.

In both methods, all correlation and all ICCs were significantly different from zero (*p* < 0.05). See Tables 4 and 5 and Figures 4 and 5.

**Table 4:** Pearson correlations and ICC_2,1_ range and value for all parameters using the new method in Study 2.


*PARAMETER*	*METHOD*	*LOWER BOUND*	*HIGHER BOUND*	*VALUE*

*Starting point*	Pearson cor.	0.719	0.864	0.803

*Boundary*	Pearson cor.	0.658	0.831	0.758

*v.true*	Pearson cor.	0.739	0.874	0.818

*v.false*	Pearson cor.	0.857	0.933	0.902

*sv*	Pearson cor.	0.923	0.965	0.948

*Starting point*	ICC_2,1_	–0.092	0.800	0.490

*Boundary*	ICC_2,1_	–0.094	0.750	0.420

*v.true*	ICC_2,1_	–0.084	0.790	0.470

*v.false*	ICC_2,1_	0.510	0.920	0.830

*sv*	ICC_2,1_	0.048	0.920	0.770


**Table 5:** Pearson correlation and ICC_2,1_ range and value for all parameters using the classic method in Study 2.


*PARAMETER*	*METHOD*	*LOWER BOUND*	*HIGHER BOUND*	*MEAN VALUE*

*Starting point*	Pearson cor.	0.255	0.579	0.431

*Boundary*	Pearson cor.	0.297	0.609	0.467

*v.true*	Pearson cor.	0.186	0.529	0.370

*v.false*	Pearson cor.	0.680	0.842	0.774

*Starting point*	ICC_2,1_	0.081	0.44	0.270

*Boundary*	ICC_2,1_	0.14	0.49	0.32

*v.true*	ICC_2,1_	0.033	0.39	0.22

*v.false*	ICC_2,1_	0.60	0.80	0.71


While comparing the two models using DIC, we found that the new method generates a better fit than the “classic” method in 58 out of the 96 ”participants” (60.41%). The summation of the DIC for each method separately yields the same conclusion, indicating a hugely lower (better) value for the new method (194,665.3) compared to the “classic” method (195,714.6), indicating decisive superiority of the new method. Considering that DIC differences of even 6 are considered to be meaningful, the current difference of 1,049.3 points probably reflects a meaningful discrepancy, even when considering DIC’s limitations.

Finally, and most importantly, we conducted a series of t-tests to compare the mean posterior values between the two groups. When using the new method, we found that the groups were significantly different from each other only in *sv*, (*t*(95) = 14.302, *p* < 0.001), in the correct direction (Mean of first group = 2.120, Mean of second group = 0.772), while the rest of the t-tests were non-significant (*p* > 0.05). In other words, the new method correctly detected the true group difference, and did not wrongly detect other differences. In a sharp contrast, the classic method (wrongly) found significant group differences in all the parameters (see supplementary materials for a full description). In other words, the classic method failed detecting the true group difference (because it existed in the fixed parameter) and, as a result, wrongly found a group difference in the remaining parameters.

### Summary

In summarizing the results of Study 2, it appears that the new method overcomes the limitations of the classical method. Specifically, when groups differ in *sv*, the classic method wrongly found differences in other parameters, while the new method accurately revealed the true differences between the groups. Additionally, the new method recovered the parameters of the individual participants successfully while employing Pearson correlation and ICC_2,1_. However, this time we found that the differences between Pearson correlation and ICC_2,1_ were substantial, suggesting that (both models) were better at recovering differences between individuals and groups than recovering absolute values. The reason for this discrepancy that was found in Study 2 but not in Study 1, remains unclear. Nevertheless, we noticed that the recovered parameter values in Study 2 were systematically higher than the real values, as in the Supplementary Materials.

It is important to emphasize that the exact value of a parameter seems to often lack practical significance, since in most cases researchers are interested in differences. This implies that the discrepancy between the Pearson correlation and the ICC_2,1_ result, while not being fully understood, does not seem to have much relevance.

Another noteworthy aspect regarding the results of Study 2 is the wide confidence interval of the ICC_2,1_, occasionally encompassing zero, despite the significance. Apparently, since the p-values and the confidence interval are calculated using different methods, these results may occur.

## Discussion

In this study, we suggested a new method to fit EAMs. In the standard usage, one must fix an individual-level parameter that sets the scale for the remaining parameters, and this setting means that we assume that participants or groups are not different from one another in this particular parameter. The problem is that this assumption might be wrong. If this assumption is violated, it makes it impossible to compare individuals or groups in the values of their freely estimated parameters. The new method solves this limitation by *de facto* fixing the hyper parameter describing the *population* level instead of fixing it at the *individual* level. We wish to emphasize that our arguments pertain to individual differences, existing group differences (such as in comparing elderly to young participants) but also to randomly assigned groups that received different treatments. This is because, unless checked, one cannot know whether the treatment has influenced the fixed parameter such that it eventually varied between groups or individuals.

More specifically, the new method is suitable for the Bayesian hierarchical version of EAM’s. Using this version, we employed the Linear Ballistic Accumulator model as a proof of concept, and defined *Mu* priors (the population mean prior) for the *sv* parameter with a *Beta* distribution with α = β = 1, i.e., a uniform distribution, with a narrow range of 0.999 to 1.001. This prior’s definition allows for individual differences in this parameter by *de facto* constraining the population mean of this parameter alone, which sets the scale for all the free parameters (aside from t0, which already has a sec. scale).

To check the applicability of the new method, we ran two parameter recovery studies. In Study 1, we found quite reasonable parameter recovery, as indicated by Pearson correlation and ICC_2,1_ between the “true” parameter values and the estimated ones. Additionally, we found that the differences between Pearson correlation and ICC_2,1_ were quite low, suggesting that the recovery was successful both in terms of stability of relative scores and in absolute agreement. The only parameter that did not recover well was *v.false*. To show that this is not a byproduct of using the new method, we ran another parameter recovery using the classical method and once again found that all parameters recovered successfully aside from *v.false*. These results confirm our suspicion that *v.false* is not so well recovered regardless of the method being used.

Study 2 addressed three shortcomings of Study 1: (a) using priors that were unrealistically close to the truth and (b) failing to demonstrate a better model fit for the new method failing to demonstrate a better model fit for the new method, (c) dis-considering of groups or condition diffidences. In Study 2, we used priors that were less close to the truth than in Study 1, compared between the methods in terms of model fit, and additionally considered group differences. The results indicate that the model of the new method was decisively superior to the classic model, and also yielded better recovery in terms of Pearson correlation, and ICC_2,1_. *Critically, while only the new method was successful in detecting true group differences, the classic method wrongly identified non-existing group differences*.

The current parameter recovery work employed the LBA, but a more widely used EAM is the DDM (Ratcliff & McKoon, 2008). When fitting the DDM, the fixed parameter is not the across-trial standard deviation of the (individual) drift-rates (which is usually fixed when fitting the LBA), but instead, the within-trial standard deviation of the drift-rate. Such within-trial variation in drift-rate is absent in the LBA, which is intentionally simplified. Nonetheless, all the current arguments remain relevant for the DDM as well, because even when using the DDM, it remains to be shown that individual and group differences in within-trial drift-rates are negligible. Such an assumption is far from being warranted given that such fluctuations may also represent neural noise and given the known meaningful individual differences in neural noise (Dinstein et al., 2015).

Although we demonstrated successful parameter recovery using the new method, this paper only provides proof of concept. More specifically, this paper provides support for the new method using (1) the Linear Ballistic Accumulation model, (2) 21 different combinations of mean parameters values that are realistic for some tasks but may not be realistic for other tasks (3) a fixed standard deviation for each parameter in each model, and (4) priors that do not differ too much from the true values – i.e. indicating a situation in which there is already quite substantial knowledge regarding the task being studied. Applying the new methods to situations that differ too much from those studied here necessitates running additional parameter recovery studies.

In summary, the benefits of the new method suggest that it should be seriously considered especially when comparing between individuals and groups because the literature implicates true individual differences in *sv*.

## Data Accessibility Statement

Parameter recovery and analysis of the parameter recovery results were conducted using R, version 4.1.2 & 4.2.2 (R Core Team, 2020). All code, data results and a working example of the new method are available here.

## Additional File

The additional file for this article can be found as follows:

10.5334/joc.394.s1 Supplementary Materials. Supplementary Materials that include additional analyses (https://osf.io/3jvsp/files/osfstorage).
