# Supplementary material for: One Standard for All: Uniform Scale for Comparing Individuals and Groups in Hierarchical Bayesian Evidence Accumulation Modeling
Source: J Cogn. 2024 Aug 16;7(1):65. doi: 10.5334/joc.394 (PMC11328677; doi:10.5334/joc.394)
Supplement: Supplementary Materials. — Supplementary Materials that include additional analyses (https://osf.io/3jvsp/files/osfstorage). [file joc-7-1-394-s1.pdf]

One Standard for All: Uniform Scale for Comparing Individuals and Groups in Hierarchical Bayesian Evidence Accumulation Modeling

Rotem Berkovich[1*], Nachshon Meiran[1]

Ben Gurion University of the Negev, Beer-Sheva, Israel

Corresponding Author: Rotem Berkovich, Ben-Gurion University of the Negev, Beer-Sheva, Israel, Tel: +972-537266209, email: rotember@post.bgu.ac.il.

Study 1

Pearson correlation and ICC

New method

In the results reported here, none of the simulated participants was excluded. We found that all correlation and all ICCs were significantly different from zero ($p < 0.05$), besides that of *v.false* whose ICC was not significant in 8 out of the 21 models. The range of values is shown in Table S1. Additionally, the Pearson correlation results are shown in Figure S1a, and the $ICC_{2,1}$ results are shown in Figure S1b.

Table S1.

Pearson correlation and $ICC_{2,1}$ range and mean for all parameters using the new method in Study 1.

Parameter	*Method*	*Lower bound*	*Higer bound*	*Mean value*
Starting point	Pearson cor.	0.755	0.898	0.839
Boundary	Pearson cor.	0.717	0.908	0.831
v.true	Pearson cor.	0.862	0.941	0.907

v.false	Pearson cor.	0.285	0.556	0.440
sv	Pearson cor.	0.871	0.960	0.924
Starting point	$ICC_{2,1}$	0.647	0.895	0.819
Boundary	$ICC_{2,1}$	0.688	0.905	0.809
v.true	$ICC_{2,1}$	0.848	0.939	0.895
v.false	$ICC_{2,1}$	0.038	0.502	0.208
sv	$ICC_{2,1}$	0.847	0.953	0.918

Figure S1

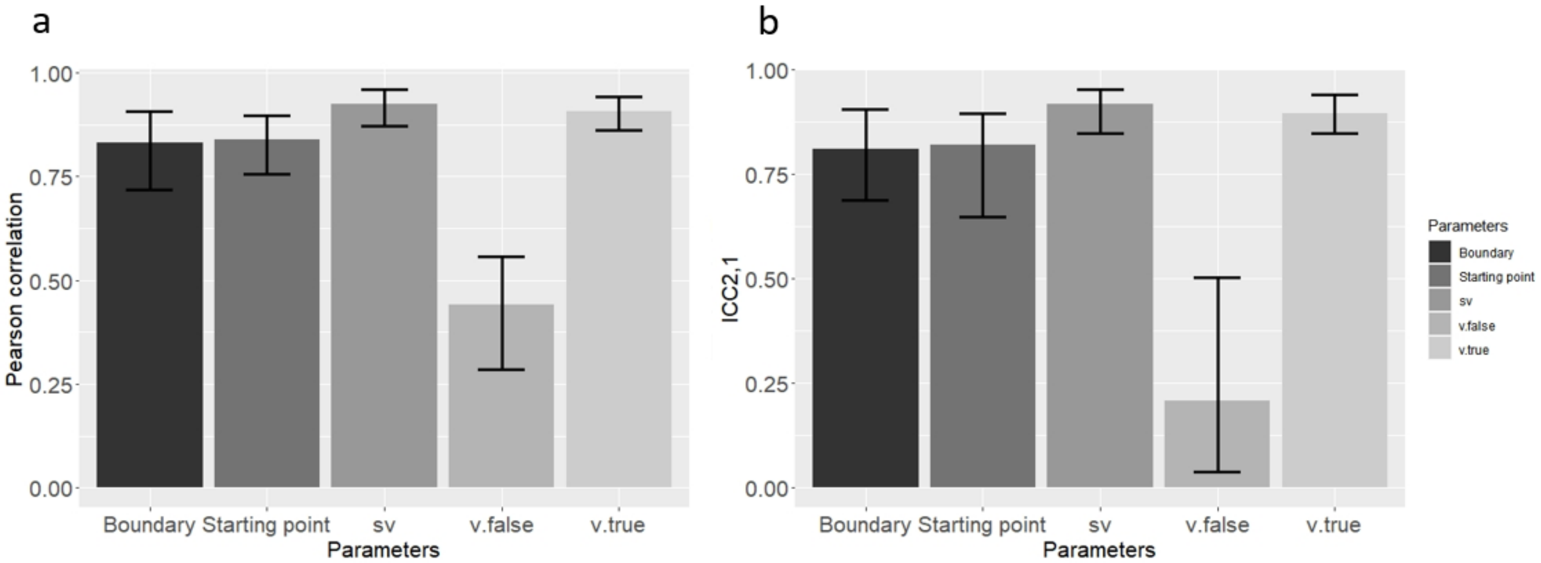

Mean (a) Pearson correlation, and (b) ICC2,1 Across the 21 Simulated Datasets (Means were computed through Fisher's Z transformation), using the new method in Study 1. Error bars represent the lower and upper range of correlations from the 21 models.

Classic method

In the results reported here, none of the simulated participants was excluded. Due to a coding error, ICC for the boundary parameter was not calculated for the entire sample. All

Pearson correlation for all parameters (except for *Boundary*) were significantly different from zero ($p < 0.05$). This time, all $ICC_{2,1}$ was significantly different from zero, except for the *v.false* that was not significant in 4 out of the 21 models. All results of this analysis are reported in Table S2 and Figure S2.

Table S2.

Pearson correlation and $ICC_{2,1}$ range and mean for all parameters using the classic method in Study 1.

Parameter	***Method***	***Lower bound***	***Higer bound***	***Mean value***
Starting point	Pearson cor.	0.780	0.920	0.881
Boundary	Pearson cor.	0.966	0.992	0.987
v.true	Pearson cor.	0.988	0.993	0.991
v.false	Pearson cor.	0.424	0.957	0.615
Starting point	$ICC_{2,1}$	0.756	0.917	0.869
v.true	$ICC_{2,1}$	0.988	0.993	0.991
v.false	$ICC_{2,1}$	0.043	0.956	0.433

Figure S2

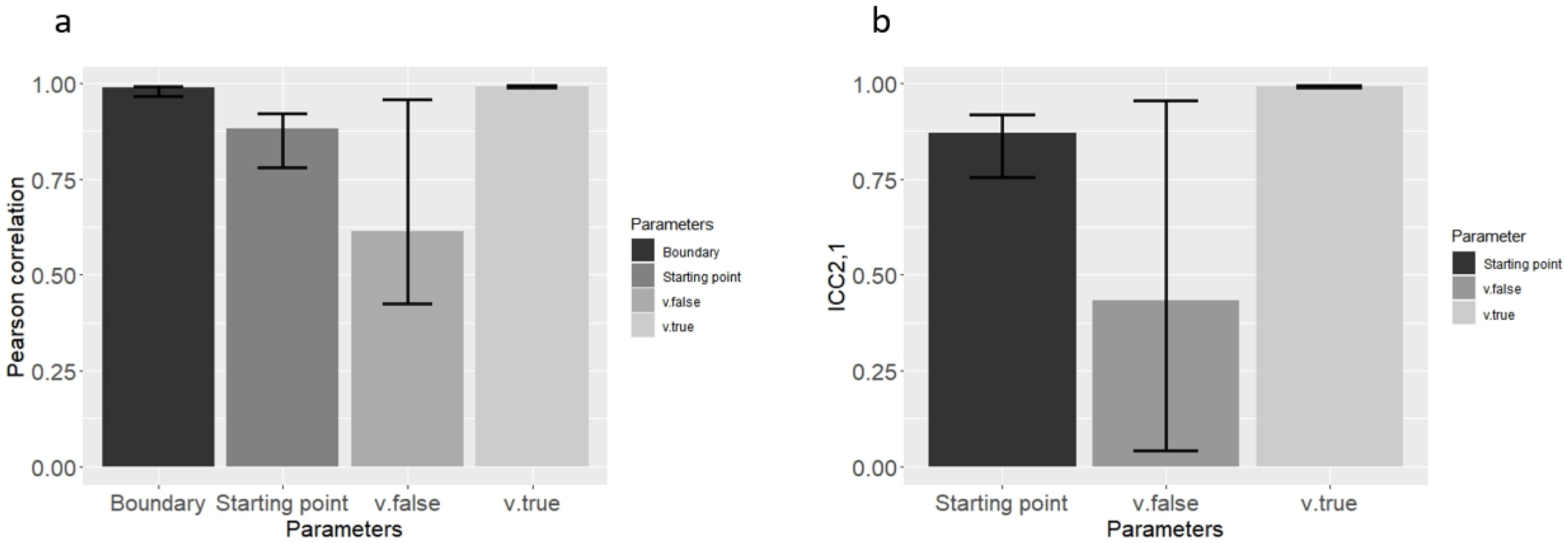

Mean (a) Pearson correlation, and (b) ICC2,1 Across the 21 Simulated Datasets (Means were computed through Fisher's Z transformation), using the classic method in Study 1. Error bars represent the lower and upper range of correlations from the 21 models.

<u>Study 2</u>

Pearson correlation and ICC

New method

In the results reported here, none of the simulated participants was excluded. We found that all correlation and all ICCs were significantly different from zero ($p < 0.05$). The values and range of the correlation and ICCs are shown in Table S3.

Table S3.

Pearson correlation and $ICC_{2,1}$ range and mean for all parameters using the new method in Study 2.

Parameter	***Method***	***Lower bound***	***Higher bound***	***Mean value***
Starting point	Pearson cor.	0.709	0.857	0.795

Boundary	Pearson cor.	0.643	0.821	0.745
v.true	Pearson cor.	0.735	0.871	0.814
v.false	Pearson cor.	0.848	0.928	0.895
sv	Pearson cor.	0.930	0.968	0.953
Starting point	$ICC_{2,1}$	-0.094	0.790	0.490
Boundary	$ICC_{2,1}$	-0.096	0.740	0.410
v.true	$ICC_{2,1}$	-0.085	0.790	0.470
v.false	$ICC_{2,1}$	0.540	0.920	0.820
sv	$ICC_{2,1}$	0.089	0.930	0.790

Classic method

In the results reported here, none of the simulated participants was excluded. We found that all correlation and all ICCs were significantly different from zero ($p < 0.05$), except for the correlation and ICC_{12} of *v.true*, that was not significantly different from zero. The values and range of the correlation and ICCs are shown in Table S4.

Table S4.

Pearson correlation and $ICC_{2,1}$ range and mean for all parameters using the classic method in Study 2.

Parameter	***Method***	***Lower bound***	***Higher bound***	***Mean value***

Starting point	Pearson cor.	0.265	0.585	0.439
Boundary	Pearson cor.	0.281	0.596	0.453
v.true	Pearson cor.	-0.053	0.332	0.145
v.false	Pearson cor.	0.675	0.839	0.769
Starting point	$ICC_{2,1}$	0.097	0.450	0.280
Boundary	$ICC_{2,1}$	0.130	0.480	0.310
v.true	$ICC_{2,1}$	-0.110	0.250	0.066
v.false	$ICC_{2,1}$	0.590	0.790	0.710

t-test analysis

The results of all t-tests are reported in Table S5.- S8.

New method

<u>Without removing participants</u>

Table S5.

t-test results for all parameters using the new method, before removing any participants from the analysis

Parameter	***t***	***df***	***p-value***	***Mean of first group (original sv=1.5)***	***Mean of second group (original sv=0.5)***

Starting point	0.416	98	>0.05	2.704	2.759
Boundary	1.473	98	>0.05	3.223	3.423
t0	0.965	98	>0.05	0.316	0.328
v.true	0.027	98	>0.05	3.437	3.433
v.false	0.922	98	>0.05	0.990	1.121
sv	14.682	98	< 0.001	2.120	0.727

After participants' removal

Table S6.

t-test results for all parameters using the new method, after removing participants from the analysis

Parameter	***t***	***df***	***p-value***	***Mean of first group (original sv=1.5)***	***Mean of second group (original sv=0.5)***
Starting point	0.563	95	>0.05	2.704	2.779
Boundary	1.140	95	>0.05	3.223	3.378
t0	1.106	95	>0.05	0.316	0.330
v.true	0.119	95	>0.05	3.437	3.455
v.false	1.015	95	>0.05	0.990	1.139
sv	14.302	95	< 0.001	2.120	0.772

Classic method

Without removing participants

Table S7.

t-test results for all parameters using the classic method, before removing any participants from the analysis

Parameter	***t***	***df***	***p-value***	***Mean of first group (original sv=1.5)***	***Mean of second group (original sv=0.5)***
Starting point	9.680	98	<0.001	1.280	3.538
Boundary	8.973	98	<0.001	1.740	3.750
t0	4.103	98	<0.001	0.301	0.515
v.true	7.432	98	<0.001	1.784	4.982
v.false	3.88	98	<0.001	0.552	1.195

After participants' removal

Table S8.

t-test results for all parameters using the classic method, after removing participants from the analysis

Parameter	***t***	***df***	***p-value***	***Mean of first group (original sv=1.5)***	***Mean of second group (original sv=0.5)***
Starting point	10.115	97	<0.001	1.280	3.594
Boundary	8.910	97	<0.001	1.740	3.756
t0	3.939	97	<0.001	0.301	0.502

v.true	10.800	97	<0.001	1.784	4.641
v.false	3.868	97	<0.001	0.552	1.198

Deviance Information Criterion (DIC)

While comparing the two models using DIC without excluding any participant, we found that the new method's model better fits the data in 62 out of the 100 ”participants” (62%), compared with the classic method model. The summation of the DIC for each method separately yields the same conclusion, indicating a hugely lower value for the new method (202,101.9) compared to the classic method (202,618.5), indicating decisive superiority of the new method.
